# Acellular Appendix Vermiform Mucinous Neoplasm

**DOI:** 10.1155/crip/7732249

**Published:** 2024-12-05

**Authors:** Panagiotis Tsikouras, Christos Tsalikidis, Efthymios Oikonomou, Maria Kouroupi, Konstantinos Nikolettos, Anastasia Bothou, Theopi Nalmpanti, Nektaria Kritsotaki, Sonia Kotanidou, Georgios Iatrakis, Nikolaos Nikolettos

**Affiliations:** ^1^Department of Obstetrics and Gynecology, Democritus University of Thrace, Alexandroupolis, Greece; ^2^Second Department of Surgery, University General Hospital of Alexandroupolis, Democritus University of Thrace, Alexandroupolis, Greece; ^3^Department of Pathology, Democritus University of Thrace, Alexandroupolis, Greece

**Keywords:** acellular appendix vermiform, diagnosis, management

## Abstract

Appendiceal neoplasms are usually asymptomatic or associated with mild, nonspecific symptoms. Due to the rarity of the disease and the lack of specific symptoms, this clinical entity escapes the diagnostic consideration of the gynecologist, when women come in with right iliac fossa pain. A case is presented of a 56-year-old woman with a mass in the right small pelvis, which was preoperatively diagnosed as originating from the ovary. An exploratory laparotomy followed in which the uterus and appendages were found to be macroscopically normal, while the mass described above came from the appendix, extended into the anatomical area of the right accessory, and was in contact with the atrophic right ovary. The appendix vermiformis was removed intact. The final pathologic examination confirmed an acellular mucinous tumor of the appendix. Accurate preoperative diagnosis of mucoceles is extremely difficult to make. The formation is discovered in a random imaging test, and the diagnosis is confirmed only intraoperatively.

## 1. Introduction

The appendix, 5–35 cm in length, is a narrow, hollow, blunt-ended tube that connects to the cecum. Its mucosa is analogous to that of the large intestine and is distinguished by the presence of columnar epithelium, neuroendocrine cells, and mucus-producing goblet cells in its cylindrical structure [1]. Neoplasms, both benign and malignant, are rarely found in the appendix, with carcinoid being more common. Their clinical picture is nonspecific, resembling acute appendicitis, which makes preoperative diagnosis extremely difficult. Other benign neoplasms of the appendix are adenomas, the most common being cystadenoma, retention cyst, and diffuse hyperplasia (hyperplastic polyp) [2, 3]. Neoplasms of the organ account for 0.4%–1% of the total number of neoplasms of the gastrointestinal system. Approximately 1% of all colon neoplasms are located in the appendix vermiform and can be found unexpectedly in any planned or emergency surgery, which includes the appendix as an exclusive or nonsurgical preparation [2, 3]. Independent risk factors for the manifestation of a primary appendiceal vermiform neoplasm are advanced age of the patient, absence of a migratory character with regard to right iliac fossa pain, and presence of an inflammatory mass on computed tomography [2, 3].

## 2. Case Report

A 53-year-old woman, Gravida IV, Para II, presented with a painful lower abdominal mass of 6 months' duration. She reported that she often went to the emergency department with the following complaints: sharp lower abdominal pain associated with anorexia, nausea, malaise, and occasionally bloody stools. Her last menstruation 6 months ago was associated with severe abdominal pain for the first 5 days. In the last 2 months, she noted dyspareunia. She had no past medical history, no systemic symptoms, and no previous operations and took no medications. On the last midcycle, the mentioned woman was hospitalized with recurrent abdominal pain of moderate to severe intensity. The gynecological examination was unremarkable except for the lower right abdominal quadrant. She had localized rebound tenderness in this area. Her blood chemistry test showed no abnormality, and the serum levels of cancer antigen 125 (CA125) and CEA were within normal range.

**Figure 1:**
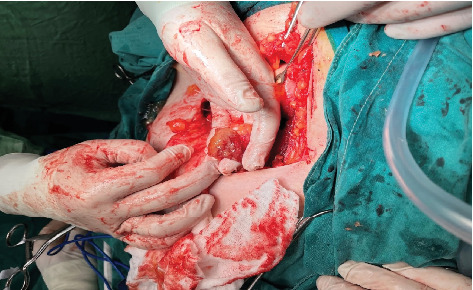
The appendix distended in saccular form.

**Figure 2:**
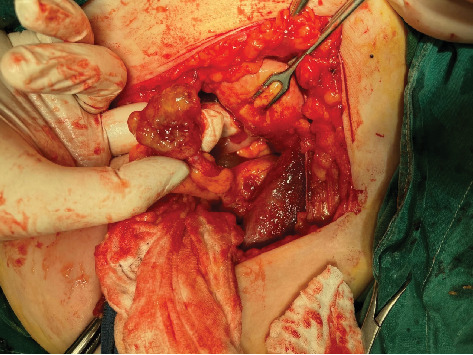
The appendix specimen shown as a gelatinous polycystic mass.

Transvaginal sonography (TVS) was performed and revealed the presence of a large inhomogeneous cystic mass 8 cm in diameter in the right ovary with numerous round, intensely hyperechoic and hypoechoic masses contained within. Doppler assessment showed the presence of normal blood flow to both ovaries. The surgery was performed by cecal adhesiolysis, followed by removal of the right adnexa, uterus, left adnexa, and the bulge of the appendix vermiform cyst as well as the solid cystic elements (Figures 1 and 2). A specific antiadhesion agent was used and placed intraoperatively into the surgical area. Particular attention was paid to carefully close the fascia and abdomen laparotomy position to avoid hernia development, incarceration of the small intestine, and local infections. A drainage was put into the Douglas pouch.

Although the intraoperative findings suggested an appendiceal mass, the surgical team decided to perform a total hysterectomy due to several factors that raised concern for a gynecologic malignancy. Preoperative ultrasound and clinical evaluation indicated the possibility of a gynecological origin for the mass, potentially involving the uterus or ovaries. Given that appendiceal and gynecological cancers can sometimes present similarly and that thorough inspection revealed possible invasion or close proximity of the mass to the reproductive organs, we opted for a more extensive resection. This approach was also intended to prevent any potential spread of malignancy, as the patient's presentation raised concerns about peritoneal dissemination.

In addition, a total hysterectomy was chosen to address any incidental or concurrent gynecologic pathology that might have been contributing to the patient's symptoms. In cases where definitive diagnosis is unclear until full pathology can be reviewed, it is sometimes necessary to err on the side of more comprehensive resection. This strategy was ultimately taken to ensure that any underlying or unrecognized gynecologic pathology would be managed appropriately in the event of malignancy confirmation, supporting an oncologically sound outcome for the patient.

The extracted organ specimens were submitted for histopathological examination and showed an essentially normal formation of the uterus and both adnexa. Postoperative histologic examination confirmed the preoperative suspicious diagnosis of appendix vermiform tumor. The histopathological examination included the following:

a. A surgical specimen of total hysterectomy 8 × 6 × 2.5 cm in dimensions and weighing 58 g, without macroscopic lesions, with the right ovary, measuring 2.3 × 1.2 × 1 cm, with a corresponding fallopian tube 3.7-cm long. The left ovary, measuring 2.5 × 1.8 × 0.8 cm, with a corresponding fallopian tube of 4 cm
b. Gelatinous composition material weighing 102 g
c. Appendix 4 cm in length, slightly dilated, with periappendiceal adipose tissue weighing 12 g. Dissections of the appendix revealed mucin in the lumen penetrating the wall up to the serosal membrane

Hematoxylin–eosin sections of the total hysterectomy specimen were without essential findings, though those of the gelatinous material showed mucin without the presence of epithelial cells. Microscopic examination of the appendiceal gelatinous lesion revealed an expansile low-grade epithelial proliferation (LAMN (low-grade appendiceal mucinous neoplasm)).

The findings describe a mucinous neoplasm of the appendix with the following characteristics.

There is a proliferation of mucinous epithelial cells that display abundant apical mucin and elongated nuclei with minimal atypia, indicating low-grade cytologic features.

Adjacent areas show some loss of crypts, which may suggest localized disruption of normal mucosal architecture.

The neoplasm has invaded the wall of the appendix, extending to and rupturing the serosal membrane, which is classified as pT4a under the TNM (tumor, node, metastasis) staging system. This level of invasion typically indicates a higher risk for complications, including potential for spread beyond the appendix.

Importantly, no epithelial cells were observed within the mucin outside the appendix, suggesting that while mucin has spread, it is free of neoplastic cells, which may reduce the risk of peritoneal dissemination associated with pseudomyxoma peritonei (PP).

**Figure 3:**
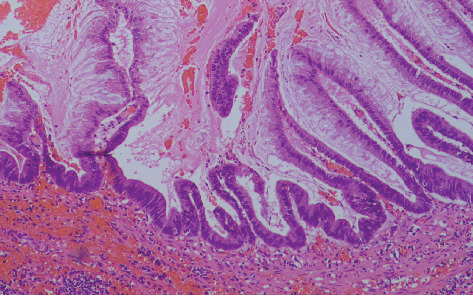
Epithelial mucinous cells with apical mucin and elongated nuclei with low-grade atypia (hematoxylin–eosin stain, ×10 HPF magnification).

**Figure 4:**
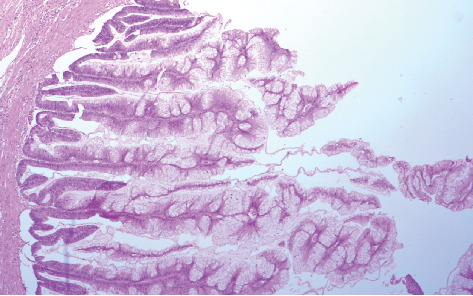
Villous proliferation mucinous epithelial cells that originate from the lumen of the appendix (hematoxylin–eosin stain, ×4 HPF magnification).

This report aligns with features seen in LAMNs, specifically due to the low-grade atypia, mucin production, and invasion reaching the serosa without extra-appendiceal neoplastic epithelial cells in the mucin (Figures 3 and 4).

The patient had an uneventful postoperative course and was discharged from the hospital 3 days after the operation. The first follow-up examination was scheduled 3 months postoperatively, and the MRI 1 year after the surgical procedure revealed no pathological findings, including local or distant metastases.

## 3. Discussion

Approximately 50% of appendiceal vermiform neoplasms present with a clinical picture of acute appendicitis and are diagnosed during histopathological examination of the surgical specimen at a rate of 0.7%–1.7%. Long-term oncological outcomes in patients are worse compared to those with colon tumor [4]. The widespread use of computed tomography has greatly increased the diagnosis of appendiceal vermiform neoplasms. The two most common malignant neoplastic tumors of the appendix are carcinoid and adenocarcinoma. Approximately 66% of malignant neoplasms of the appendix vermiform involve carcinoid (the most common type), 20% involve PP, and the rest involve histological variants of adenocarcinoma (adenocarcinoma, adenocarcinoid, and mucinous adenocarcinoma) [5].

Mucous neoplasms are found several times more often, compared to the intestinal type of adenocarcinoma. Mucous neoplasms of the appendix are mostly not malignant. In case of their rupture, they can lead to intraperitoneal spread of the tumor and manifestation of PP.

LAMNs occur most commonly in women during the sixth decade of life. When the disease is only at the appendix, it presents with acute appendicitis-like symptoms. If it is disseminated, it presents as abdominal or ovarian masses or PP is characterized morphologically as well-differentiated adenomas, which may exhibit malignant behavior outside the appendix. Twenty-five percent to 50% of LAMNs are an incidental finding [6, 7]. Some researchers have suggested the term LAMN for tumors that exhibit low-grade cytologic atypia and minimal architectural complexity, regardless of whether the tumor is limited to the mucosa (referred to as mucinous adenoma) or has extended beyond the appendix with spread of neoplastic epithelium (termed LG-HR) [6].

They are divided into two main classes, LAMN I and LAMN II. LAMN I type occurs in younger patients. The tumor is located within the lumen of the appendix. It rarely shows a tendency to progress. LAMN II type usually occurs in older patients. The guidelines for its therapeutic management include prophylactic cytoreductive surgery combined with hyperthermic intraperitoneal chemotherapy, resection of the major epiglottis, splenectomy, resection of the peritoneum in the right–left upper quadrant of the abdomen and pelvis, resection of the minor epiglottis, cholecystectomy, resection of the rectosigmoid, and antrectomy of the stomach. If the mucinous lesion is confined to the appendix, it is considered cured only by resection. Lesions with acellular mucin outside the organ have low risk for recurrence or progression, occurring in about 4% of cases [8]. Right hemicolectomy should not be performed in patients with LAMN because the occurrence of lymphovascular spread is uncommon.

PP is a rare case characterized by the presence of mucous ascites (“jelly belly”) and peritoneal implants. This is the prevalent occurring clinical entity, resulting from perforation of an appendiceal vermiform neoplasm. It is characterized by an accumulation of mucus within the peritoneal cavity (adenomucinosis), which flows continuously and in a specific manner into the peritoneal cavity and pelvis [9–12].

In the case in which there are malignant cells within the mucous ascites (cellular mucus), PP occurs in 33%. In the absence of malignant cells or neoplastic epithelium within the mucous ascites (acellular mucus), PP develops in 4%.

PP syndrome is characterized by the presence of appendiceal vermiform neoplasms with minimal invasiveness, which tend to be widely distributed on the peritoneal surface. Liver or lymphatic metastases are uncommon. Peritoneum and hollow viscera resection operations are aimed at removing all macroscopically visible foci and achieving complete cytoreduction. The most common clinical finding in men and women with PP syndrome is a progressively increasing abdominal circumference. In the female population, the second most common clinical finding is an ovarian tumor, most commonly located in the right ovary [8–10]. The third most common clinical finding in both sexes is the appearance of appendicitis.

Intestinal-type adenocarcinoma of the appendix is the most common histological type of primary appendix cancer accounting for 60% of cases but represents < 0.5% of gastrointestinal cancers. Their clinical occurrence is similar to that of appendicitis. Mucinous adenocarcinoma (mucinous adenocarcinoma) concerns 10%–15% of colon cancers, with a more frequent location in the right colon and an average age of onset of 60 years. The impact on the female sex is smaller. It has a similar prognosis to colon adenocarcinoma. The treatment of all benign neoplasms is simple appendectomy.

It is worth emphasizing that malignant neoplasms of the appendix vermiform are rare, the majority of them found incidentally in appendectomy preparations, usually for acute appendicitis and the most common of these are carcinoid. The medical history and the gynecological and sonographic examination are of great importance for the prompt diagnosis of women with appendix tumor. The aim of this case presentation is to emphasize that the further treatment of malignant neoplasms after appendectomy is not uniform and depends on the size, location, and histological type of the neoplasm as well as the existence of nonmetastatic disease [9, 10].

Finally, the vigilance of the clinician is necessary in algae of the right iliac fossa that are treated conservatively. Especially in repeated episodes, additional diagnostic testing is necessary so that neoplasms in the area do not spread. Close surveillance for patients with localized periappendiceal disease following initial surgery should be under concern.

## Data Availability

All data supporting the findings of this manuscript are readily available.
